# Prevalence of Psychoactive Drug Use among Medical Students in a Medical College of Nepal

**DOI:** 10.31729/jnma.5237

**Published:** 2020-10-31

**Authors:** Jyoti Tara Manandhar Shrestha, Saurabh Tiwari, Dilip Kumar Kushwaha, Pratigya Bhattarai, Risu Raj

**Affiliations:** 1 Department of Pharmacology, Kathmandu University School of Medical Sciences, Dhulikhel, Nepal; 2 Kathmandu University School of Medical Sciences, Dhulikhel, Nepal

**Keywords:** *alcohol*, *medical student*, *psychoactive drugs*, *substance abuse*

## Abstract

**Introduction:**

Psychoactive drug is a worrisome and emerging global problem. This is a disturbing matter, especially in the case of medical students, as it affects not only their health and academic performance alone but their clinical efficiency as well. This study aims to determine the prevalence of psychoactive drug use among medical students in a medical college in Nepal.

**Methods:**

A descriptive cross-sectional study was conducted after receiving ethical clearance from the Institutional Review Committee (Ref: 258/19) among undergraduate medical students from December 2019 to June 2020. Convenience sampling was used to collect data. Data analysis was done in the Statistical Package for Social Sciences. Point estimate at 95% confidence interval was calculated along with frequency and proportion for binary data.

**Results:**

The prevalence of psychoactive drug abuse was found to be 76 (44.2%) [CI = 43.6%-44.8%]. The study showed males 59 (59%) were more indulged in abuse than females 17 (23.6%). Alcohol 72 (41.86%) was the most commonly used, then was tobacco 24 (13.95%) followed by cannabis 17(9.88%). Only two students were sedative and opioid abusers. Pleasure 38 (31.70%) and experimentation 29 (24.20%) were the two major causes of substance abuse. Tobacco was used more frequently 14 (58.33%) used daily and found to have more financial and health-related issues in the last three months.

**Conclusions:**

Even almost half of the students were using some form of psychoactive drugs, the majority of them were occasional users. Proper counseling needs to be done to address this problem. Further study should be conducted to address the influencing factors and adverse outcomes.

## INTRODUCTION

Substance abuse is an ongoing global health concern. Medical students are at a significant risk of developing addiction due to easily accessible psychoactive drugs, strenuous work, and relative isolation from social life. Unquestionably, addiction is a grave issue amongst medical students posing threat to their prestige and career.^1,2^ Moreover, the negative impact on their academic and clinical performance affects the health outcomes of patients. Hence, the lifestyle and behavior of medical students need to be studied carefully.^3^

College students being the prime target are likely to start substance abuse and are most likely to continue. Research shows the estimated prevalence rate of substance abuse among students is around 20 to 40 percent worldwide.^4,5^ Nepal shows dreadful conditions with the rate ranging from 50 to 60 percent.^3,6,7^

This research is intended to determine the prevalence of psychoactive drug use among medical students in a medical college in Nepal.

## METHODS

A descriptive cross-sectional study conducted in Kathmandu University School of Medical Sciences (KUSMS) from December 2019 to June 2020 commenced after ethical approval from the Institutional Review Committee of KUSMS (Ref: 258/19). Students from first to the fourth year enrolled under the MBBS program were eligible and thus involved in the study after obtaining their written informed consent whereas foreign students were excluded.

The sample size was calculated as follows,

$$\begin{array}{ll} \text{n} & \text{= }{\text{Z}}^{\text{2}}\times \text{p}\times \text{(1}-\text{p)}/{\text{e}}^{\text{2}}\\ & \text{= }{\left(1.96\right)}^{\text{2}}\times 0.5\times 0.5/{\left(0.08\right)}^{\text{2}}\\ & \text{= }150 \end{array}$$

Where,

n = required sample size
Z = 1.96 at 95% Confidence interval (CI)
p = prevalence, 50%
e = margin of error, 0.08

Convenient sampling was done. A modified version of the World Health Organization - Alcohol, Smoking, and Substance Involvement Screening Test version 3.0 questionnaire was used to collect responses from students.^8^

Data of 172 students were thoroughly entered, descriptive analysis was done using IBM SPSS version 25. Descriptive parameters such as frequency and percentage were calculated and depicted in tables and figures.

## RESULTS

The overall prevalence rate of the psychoactive drug was 76 (44.2%) [CI = 43.6%-44.8%]. Among 172 students, 100 (58%) were males and 72 (42%) were females. The mean age (±SD) of the participant was 20.01±1.576 (Table 1).

**Table 1:** Psychoactive drug use among gender and years of study in medical school.

Variables		Frequency n (%)
Gender	Male	59 (59.00)
	Female	17 (23.6)
Year	First year	16 (36.4)
	Second year	16 (38.1)
	Third year	20 (46.5)
	Fourth year	24 (55.8)

Alcohol, used by 72 (41.86%) of students was the most common, followed by tobacco products and cannabis, used by 24 (13.95%) and 17 (9.88%) respectively. Only two of the students reported the use of opioids and sedatives (Table 2).

**Table 2:** Percentage of psychoactive drugs used according to gender.

Variables	Frequency n (%)	Male n (%)	Female n (%)
Tobacco	24 (13.95)	23 (95.8)	1 (4.2)
Alcohol	72 (41.86)	55 (76.4)	17 (23.6)
Cannabis	17 (9.88)	16 (94.1)	1 (5.9)
Opioids, sedatives	2 (1.16)	2 (100)	-

Moreover, opioids and sedatives were used in conjunction with alcohol, tobacco products, and cannabis. About one-third of students (31.58%) reported that they used more than one psychoactive substance (Table 3).

**Table 3:** Polydrug usage patterns.

Psychoactive drug	Frequency n (%)
Alcohol, tobacco, and cannabis	13 (17.1)
Tobacco and alcohol	7 (9.21)
Alcohol and cannabis	4 (5.26)
Alcohol only	48 (63.16)
Tobacco only	4 (5.26)

None of the students reported the use of cocaine, amphetamines, inhalants, and hallucinogens. Most of the students were occasional users but about two-third (58.33%) of tobacco consumers are found to be using it regularly. About one-fourth (23.6%) of the students started using psychoactive drugs after joining medical school.

The causes were followed by frustration or relieving stress, peer pressure, and social obligation (Table 4).

**Table 4:** Cause of psychoactive drug abuse.

Variables	Frequency n (%)
Academic pressure	6 (5.0)
Peer pressure	16 (13.3)
Frustration/ stress relief	16 (13.3)
Advertisement	1 (0.8)
Pleasure	38 (31.7)
Experimentation	29 (24.2)
Feeling of compulsion	4 (3.3)
Social Obligation	10 (8.3)

Moreover, 3 (17.65%) of cannabis users had tried to cut off the substance use in the last three months and friends and relatives expressed their concern to 4 (23.53%) of the participants. Pleasure and experimentation were found to be a major cause of substance abuse. Frustration, peer pressure, and social obligation were also among the notable causes of substance abuse.

Regarding the consequences of drug use, the majority of the students did not face any consequences in the social, legal, and health aspects of their life. Some of the drug users skipped classes once or twice leading to academic deterioration. However, some tobacco users reported monthly health issues 1 (4.17%) and weekly financial issues 3 (12.5%), none of which were observed among alcohol and cannabis users.

## DISCUSSION

Substance abuse (tobacco, alcohol, prescribed and illicit drugs) is widely recognized as a critical area when it comes to medical students. Psychoactive drugs excluding caffeine were taken into account to determine prevalence, pattern and to shed light on relatively unexplored impacts on health, education, social life, legal and financial aspects in medical undergraduate students of different academic stages.

The overall prevalence of psychoactive drugs used among the medical students of KUSMS was found to be 76 (44.2%). This rate is quite high as compared to a study conducted by Arora, et al. which showed a prevalence rate of 24.2%.^9^ However, various studies conducted in Nepal shows higher prevalence compared to our study.^3,6,7^ The study showed that there was a higher frequency of drug use in male than in the female. Jaiswal, et al. stated no significant differences concerning gender.^10^ The reason behind this, maybe due to our society giving more liberty to men than women.

Alcohol 42 (41.86%) was the most commonly abused substance, followed by tobacco 24 (13.95%) and then cannabis 17 (9.88%). In comparison to the research shown by Khanal, et al., the result was reasonably similar in which the use of alcohol, tobacco, and cannabis was 57.6%, 27.6%, and 12.8% respectively.^7^ A similar pattern was reported by various studies.^7,10,11^ However, a study by Jamshid, et al. in Iran showed tobacco was the most commonly used substance as alcohol is forbidden in that region.^12^ Hence, these differences in prevalence and pattern might be accounted to different socio-cultural scenarios.

Tobacco was found to be consumed more frequently than alcohol and cannabis as the result showed that two-third (62.5%) of tobacco users were using it at least once a week. Polydrug users were approximately 32%. In contrast, a study conducted by Roy, et al. reported the use of approximately 82% of students as poly-drug users.^3^ Prevalence of poly-drug use reported in our study is far less than shown by Roy, et al. This arises a cause of concern, as poly-drug might potentiate adverse effects.

We found that medical school is not a typical place to begin alcohol and tobacco. The study suggests that a pattern of alcohol and tobacco use was established before joining the medical school, whereas, use of cannabis, opioids, and sedatives were started after joining the medical school. Pleasure (31.7%) was one of the major causes of drug abuse experimentation (24.2%) being the second-most. The third major reason was frustration (13.3%). In the case of alcohol, peer pressure and social obligation were found to have a huge role. Similarly, Khanal, et al. reported experimentation (42.3%), stress (19.5%), and pleasure (15.4%) as principal causes for the abuse.^7^ However, Budhathoki, et al. suggested pleasure (40.9%), irresistible desire (36.5%), and academic stress (20.4%) as a major culprit for tobacco use. The result illustrates the consumption of alcohol starts as a social obligation; “Matawali”^12^ category of people in which alcohol is socially acceptable, or might also be a result of an advertisement, peer pressure, or academic pressure. Once started with alcohol, other drugs are consumed as of curiosity or pleasure or to ward off psychological stress. Friends and relatives seemed to discourage cannabis use. They have tried more in abstaining from cannabis use to a greater extent than tobacco and alcohol.

Most of them reported they had not faced any consequences. Though it was found that tobacco users faced more health issues and few of them reported facing health-related issues monthly. However, Budhathoki, et al. reported about one-fifth of alcohol users faced medical issues.^6^ Some tobacco users reported having financial issues weekly as well. Only four students reported deterioration in academic performance after starting to consume substances. None of the students reported any impact related to legal and social issues. An increasing trend in substance use was seen as students climb the academic ladder. The consumption frequency as well as the prevalence of poly-drug reflects the amount of stress in students due to academic pressure. It is therefore important to sensitize the students about the harm and intervention during the early phase of medical life. This seems more crucial to prevent this awful situation before they become a qualified doctor.

This study was carried out in a single institution with a small sample size. The information provided by the participants was based on the events of the last three months, so recall bias might have hampered outcomes of the study. Thus, it cannot be generalized to the general population. We did not collect the data regarding knowledge, attitude, and practice of the subjects regarding substance use and there is no data taken on substance dependence. The use of psychoactive substance use among medical students itself is a sensitive issue, there are chances that many of the study participants may have denied using the psychoactive substances at any point in time. Therefore, we cannot rule out underreporting of substance use as a result of reporting bias, recall bias, and social desirability bias. Despite these limitations, being able to collect data anonymously on this sensitive issue in a medical profession is the strength of this study.

## CONCLUSIONS

The study showed that there was a significant increase in the use of substances among the medical students which can be considered to be a worrisome matter. Since psychoactive drug use in medical students affects the health and well-being of an upcoming doctor, and to a greater extent affects the health of the patient, therefore, it is necessary to adopt effective and suitable counseling measures promptly.
